# Safe contact-based robot active search using Bayesian optimization and control barrier functions

**DOI:** 10.3389/frobt.2024.1344367

**Published:** 2024-04-29

**Authors:** Frederik Vinter-Hviid, Christoffer Sloth, Thiusius Rajeeth Savarimuthu, Iñigo Iturrate

**Affiliations:** ^1^ ROPCA ApS, Odense, Denmark; ^2^ SDU Robotics, Maersk McKinney Moller Institute, University of Southern Denmark, Odense, Denmark

**Keywords:** control barrier function, Bayesian optimization, active search, autonomous ultrasound scanning, robot force control

## Abstract

In robotics, active exploration and learning in uncertain environments must take into account safety, as the robot may otherwise damage itself or its surroundings. This paper presents a method for safe active search using Bayesian optimization and control barrier functions. As robot paths undertaken during sampling are continuous, we consider an informative continuous expected improvement acquisition function. To safely bound the contact forces between the robot and its surroundings, we leverage exponential control barrier functions, utilizing the derivative of the force in the contact model to increase robustness to uncertainty in the contact boundary. Our approach is demonstrated on a fully autonomous robot for ultrasound scanning of rheumatoid arthritis (RA). Here, active search is a critical component of ensuring high image quality. Furthermore, bounded contact forces between the ultrasound probe and the patient ensure patient safety and better scan quality. To the best of our knowledge, our results are both the first demonstration of safe active search on a fully autonomous robot for ultrasound scanning of rheumatoid arthritis and the first experimental evaluation of bounding contact forces in the context of medical robotics using control barrier functions. The results show that when search time is limited to less than 60 s, informative continuous expected improvement leads to a 92% success, a 13% improvement compared to expected improvement. Meanwhile, exponential control barrier functions can limit the force applied by the robot to under 5 N, even in cases where the contact boundary is specified incorrectly by −1 or +4 mm.

## 1 Introduction

Active learning, along with related problems such as active object exploration and localization, find widespread applications across various robotic domains. From mobile robot navigation (Lluvia et al., 2021) to grasping (Kroemer et al., 2010) to object and scene reconstruction (Jamali et al., 2016), many robotic problems involve variations of the exploration–exploitation dilemma, where an autonomous system must simultaneously regress and optimize an unknown or uncertain function.

Several methods have been proposed to tackle the above problems, including many based on deep learning (Ren et al., 2021). For the low-data regime, which is often the case for online learning in robotics, the most common are methods based on Gaussian process regression (Jamali et al., 2016; Yi et al., 2016; Driess et al., 2019; De Farias et al., 2021) and Bayesian optimization (Nogueira et al., 2016; Goel et al., 2022; Roveda et al., 2022; Berkenkamp et al., 2023). Koopman operators have also been suggested for the case of learning the nonlinear dynamics of a physical system for control (Abraham and Murphey, 2019), but this method is only applicable to learning dynamics and not to a more general function approximation problem.

As active exploration is, by definition, an uncertain process, a key consideration is the assurance of safety despite operating in fully or partially unknown environments. Safety can be handled algorithmically in the learning process by considering, for example, the uncertainty in a Gaussian process regression (Turchetta et al., 2019). However, here we are more interested in methods that consider the safety of the controlled system, that is, the robot, as a query deemed safe algorithmically can still result in an unsafe robot action due to uncertainty in the control and environmental dynamics. Various methods have been proposed, such as safe control based on energy functions (Pandya and Liu, 2022), safe model predictive control (Koller et al., 2018), and Bayesian meta-learning through alternating sequential optimal control problems for exploration and exploitation (Lew et al., 2022). A prominent approach to safety is control barrier functions (CBFs), a framework for ensuring the safety of nonlinear control affine systems (Ames et al., 2014; Romdlony and Jayawardhana, 2014; Ames et al., 2019). The resulting equations can be solved using quadratic programming, making the implementation feasible in control loops. The methodology has been expanded to high relative degree systems, making it generally applicable (Nguyen and Sreenath, 2016). Model uncertainty has been considered in CBFs in terms of parametric uncertainty (Cohen and Belta, 2022) and non-parametric uncertainty (Castañeda et al., 2021). These works ensure that safety can be guaranteed despite having a bounded model uncertainty.

In this paper, we consider the combination of the above two problems, namely, safe active exploration, in the context of an autonomous robot system for ultrasound (US) scanning of rheumatoid arthritis (RA). US imaging is a popular method for diagnosis and monitoring of RA and various other diseases, as it allows inspection of tissue and joint structures at low cost and without the use of radiation. The use of ultrasound does, however, rely on trained professionals with limited availability, so automation of the process has high potential value.

Active exploration is a major component of US scanning for RA and is, in fact, how the procedure is performed manually by trained professionals. During the procedure, the probe is placed in contact with joints in the patient’s hand, allowing the subsurface structures to be inspected on a connected monitor. The professional must ensure that contact between the probe and the patient’s hand is maintained and find an optimal placement in order to capture potential disease activity. When an appropriate area has been found, the probe is kept stationary, and Doppler mode imaging is used to assess the flow of synovial fluid in the joint cavity, indicating inflammation. Correct placement of the probe will ensure high-quality scans. Different approaches have been proposed to optimize the ultrasound image quality. A commonly used method is based on ultrasound confidence maps (Karamalis et al., 2012; Chatelain et al., 2017; Jiang et al., 2020) that can be used to detect if contact is poor and adjustments to the probe position must be made. Force-based methods have also been explored to optimize the image quality by attempting to estimate the surface normal and position of the probe accordingly (Jiang et al., 2021a). As these methods do not consider the structure of tissues in the image but only the overall quality, they are not suited for RA, where the structure is vital. Bayesian optimization based on segmented ultrasound images is proposed by Goel et al. (2022). This allows certain structures in the image to be optimized efficiently, and as the optimization is guided by a statistical model, uncertainties are handled implicitly. While the above methods consider the positioning of the probe, limiting the maximum applied force is also critical. First, the contact force must be kept low to ensure the flow of synovial fluid is not blocked by the pressure (Möller et al., 2017). Second, patients with high levels of disease activity will experience pain from excessive pressure.

To address the above use case, we propose to draw from the wider active exploration and safe control literature. To determine the probing location, we use Bayesian optimization, similar to Goel et al. (2022), but make use of a classifier trained to estimate the US image quality instead of a segmentation model. This is done because assessing the quality of an ultrasound image for RA is a complex task requiring the correct placement, appearance, and relative size of different tissue types. We extend the method by considering the ability to sample images continuously while positioning the probe on the patient, leading to a more efficient search. To establish and maintain contact with the patient’s hand, we use direct force control and add an additional level of safety in the form of a high-order CBF to bound the contact forces below a safe threshold.

Our main contributions are as follows:

• We present, to the best of our knowledge, the first results on CBFs applied to safe force control in the medical domain of a real (not simulated) robotic system in a laboratory setting.
• We present a method for autonomous robot scanning of RA. While research has been performed on autonomous US scanning for various applications such as scanning of vessels (Virga et al., 2016; Jiang et al., 2021b) and breasts (Welleweerd et al., 2020), to the best of our knowledge, no previous work within autonomous scanning for RA exists.
• Additionally, a minor contribution is the extension of informative continuous expected improvement (ICEI) as an acquisition function for Bayesian optimization to the context of robot motion planning.

## 2 Problem formulation

We consider two sub-problems in the context of ultrasound scanning for RA:

Problem 1 (Active search). This problem is concerned with finding areas of high ultrasound image quality in the presence of uncertain measurements. We consider the search problem as finding a global maximum ***p***_*opt*_ of an unknown underlying function *f*(***p***) with uncertainty *ϵ* where ***p*** is a subset of the Cartesian space.

$$p_{\mathrm{opt}}=\underset{p\in R^{n}}{\arg}\max f\left(p\right)+\epsilon .$$ (1)

Function estimates are achieved using a classifier trained on ultrasound images. It is assumed that a starting point is given from external sensing, such as a RGB-D camera combined with a method of estimating joint locations in a RGB image. Solutions for this exist, at least for simple, well-lit scenes (Zhang et al., 2020).

**FIGURE 1:**
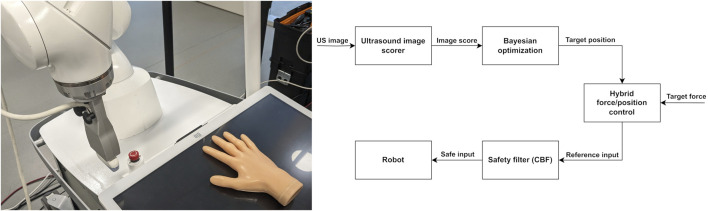
Left, the experimental setup for autonomous US scanning of RA. Right, a flowchart of the proposed system for active search for high-quality US images while achieving bounded contact force.

Problem 2 (Bounding the contact force). This problem consists of ensuring bounded contact force with the patient despite uncertainties in the patient’s position. A robot manipulator with an ultrasound probe mounted—as seen in Figure 1—is employed. The robot is controlled at the joint position level. We consider the problem of finding a safe control input *u* ^*^ given a potentially unsafe nominal control input *u*_no_ that minimizes the differences between *u* ^*^ and *u*_no_. Safety is defined in terms of bounded contact force *f*_*c*_ ≤ *f*_max_, where *f*_c_ and *f*_max_ are the contact force and maximum allowed force, respectively.

## 3 Preliminaries

This section will present theoretical background on Gaussian process regression and Bayesian optimization, which are used as the basis of our solution to Problem 1, and exponential control barrier functions, which are used as part of our solution to Problem 2.

### 3.1 Gaussian process regression

Gaussian process regression (GPR) allows unknown functions, along with their uncertainty, to be estimated based on limited samples (Rasmussen and Williams, 2005). A Gaussian process (GP) is a distribution over functions and is defined fully by a mean function *m*(***p***) and a covariance function *k* (***p***, ***p*** ′). It is assumed that the vector input $p\in R^{d}$ is related to the scalar output *y* by the function *f* (⋅) with the following relationship: *y* = *f*(***p***) + *ϵ*, where $\epsilon ∼N\left(0,\sigma_{\epsilon }^{2}\right)$ . The training input is defined as **P** with ***y*** being the associated targets. Together, this is denoted as $D:\left\{\left(p_{1},y_{1}\right),\left(p_{2},y_{2}\right),\ldots ,\left(p_{n},y_{n}\right)\right\}$ . Observations ***y*** and discrete function values to be predicted f_*_ then follow a multivariate normal distribution:

$$\left[\begin{array}{c} y\\ f_{*} \end{array}\right]∼N\left(\left[\begin{array}{c} m\left(P\right)\\ m\left(P_{*}\right) \end{array}\right],\left[\begin{array}{cc} K_{\epsilon } & K\left(P,P_{*}\right)\\ k\left(P_{*},P\right) & K\left(P_{*},P_{*}\right) \end{array}\right]\right),$$ (2)

where $K_{\epsilon }=K\left(P,P\right)+\sigma_{\epsilon }^{2}I$ , with $\sigma_{\epsilon }^{2}$ being the inherent noise on the measurements—allowing optimization under uncertainty—and

$$K\left(P,P^{\prime }\right)=\left[\begin{array}{cccc} k\left(p_{1},p_{1}^{\prime }\right) & k\left(p_{1},p_{2}^{\prime }\right) & \ldots & k\left(p_{1},p_{k}^{\prime }\right)\\ k\left(p_{2},p_{1}^{\prime }\right) & k\left(p_{2},p_{2}^{\prime }\right) & \ldots & k\left(p_{2},p_{k}^{\prime }\right)\\ \vdots & \vdots & \ddots & \vdots \\ k\left(p_{k},p_{1}^{\prime }\right) & k\left(p_{k},p_{2}^{\prime }\right) & \ldots & k\left(p_{k},p_{k}^{\prime }\right) \end{array}\right].$$ (3)

The mean vector associated with f_*_ is then

$${\bar{f}}_{*}=m\left(P_{*}\right)+K\left(P_{*},P\right)K_{\epsilon }^{-1}\left(y-m\left(P\right)\right)$$ (4)

and the covariance is given by:

$$\text{cov}\left(f_{*}\right)=K\left(P_{*},P_{*}\right)-K\left(P_{*},P\right)K_{\epsilon }^{-1}K\left(P,P_{*}\right).$$ (5)

The estimate of the standard deviation along the estimated function is then the diagonal of cov (f_*_) and is denoted *σ*_*f*_(***p***). The mean function is often set to zero unless some underlying information about the process exists. The covariance function is generally defined such that points near each other are more correlated. A common choice is the Matern class of covariance functions. This class of functions leads to different levels of differentiability and, thus, smoothness of *f*(***p***) based on a smoothness parameter *ν*. For $\nu =\frac{3}{2}$ , the Matern kernel is

$$k_{\nu =\frac{3}{2}}\left(p,p^{\prime }\right)=\left(1+\sqrt{3}d\left(p,p^{\prime }\right)\right)\exp\left(-\sqrt{3}d\left(p,p^{\prime }\right)\right),$$ (6)

where *d* (***p***, ***p*** ′) is the Euclidean distance between ***p*** and ***p*** ′ scaled by a diagonal matrix **Θ**:

$$d=\left(p-p^{\prime }\right)\Theta^{-1}\left(p-p^{\prime }\right),$$ (7)

$$\Theta =\text{diag}\left(l_{1},l_{2},\ldots ,l_{d}\right)=\text{diag}\left(\theta \right),$$ (8)

with $\theta ={\left[\begin{array}{ccc} l_{1} & l_{2} & \ldots l_{d} \end{array}\right]}^{T}$ as the length scales of the function. The length scales encode how rapidly *f* (⋅) changes along each dimension. These are considered free parameters that must be determined from the training data. This is done by maximizing the marginal log-likelihood of the training examples given the parameters.

### 3.2 Bayesian optimization

Bayesian optimization provides a framework for maximizing functions that are costly to evaluate, where uncertainty is present, and where following the gradient is infeasible (Shahriari et al., 2016). The method consists of three elements: a statistical surrogate model, an acquisition function, and a stopping criterion. A common choice for the surrogate model is Gaussian processes, as defined above. The acquisition function *α*(⋅) determines where to sample next in the search space based on the surrogate model by balancing exploration and exploitation:

$$p_{n+1}=\underset{p\in R^{d}}{\arg}\max\alpha \left(p;D\right).$$ (9)

A common choice for the acquisition function is expected improvement (EI) (Mockus et al., 1978). EI maximizes the expected gain over the previous best sampled score, *f* ^*^.

$$EI\left(p\right)≔E\left[\max\left(\left(f\left(p\right)-f^{*}\right),0\right)\right],$$ (10)

EI can be calculated analytically as

$$EI\left(p\right)=\left\{\begin{array}{cc} \left({\bar{f}}_{*}\left(p\right)-f^{*}\right)\Phi \left(\frac{{\bar{f}}_{*}\left(p\right)-f^{*}}{\sigma_{f}\left(p\right)}\right)+\sigma_{f}\left(p\right)\phi \left(\frac{{\bar{f}}_{*}\left(p\right)-f^{*}}{\sigma_{f}\left(p\right)}\right)\; & \text{if}\;\;\sigma_{f}\left(p\right)>0\\ 0\; & \text{if}\;\;\sigma_{f}\left(p\right)=0 \end{array}\right.,$$ (11)

where Φ(⋅), *ϕ*(⋅) are the cumulative distribution function (CDF) and the probability density function (PDF), respectively.

### 3.3 Control barrier functions

Control barrier functions provide a framework for ensuring system safety (Ames et al., 2014). Throughout this section, a nonlinear control affine system of the following form is considered:

$$\dot{x}=f\left(x\right)+g\left(x\right)u,$$ (12)

where $f:R^{n}\to R^{n}$ and $g:R^{n}\to R^{n\times m}$ are local Lipschitz functions, with $x\in X\subset R^{n}$ and $u\in U\subset R^{m}$ being the admissible states and control inputs, respectively. The goal of the CBF is to keep the system in a safe region of the state space. This safe region C is defined based on a continuously differentiable function $h:X\subset R^{n}\to R$ as

$$\begin{array}{ll} C\; & =\;\left\{x\in X\;|\;h\left(x\right)\geq 0\right\},\\ \partial C\; & =\;\left\{x\in X\;|\;h\left(x\right)=0\right\},\\ \text{Int}\left(C\right)\; & =\;\left\{x\in X\;|\;h\left(x\right)>0\right\}. \end{array}$$ (13)

The system is thus safe if a control law input ***u*** leads to the scalar function $h\left(x\right)\geq 0\;\forall t\wedge \forall x_{0}\in C$ . Equivalently, this can be described as the set C being rendered forward invariant by the control law. When the relative degree *r* of the system with respect to *h* is larger than one, higher-order CBFs can be used to render a set C safe. The relative degree of a continuously differentiable function *h*(***x***) with respect to System (12) is the number of times it can be differentiated along (12) before the control input ***u*** explicitly shows. Exponential control barrier functions (ECBFs) are a class of higher-order CBFs (Nguyen and Sreenath, 2016). This formulation is based on calculating higher-order time derivatives of *h*(***x***). The *r* ^th^ time derivative is

$$h^{\left(r\right)}\left(x,u\right)=L_{f}^{r}h\left(x\right)+L_{g}L_{f}^{r-1}h\left(x\right)u,$$ (14)

where $L_{f}^{r}h\left(x\right)$ is the *r* ^th^ Lie derivative of *h* along *f*. ECBFs are defined based on a series of integrators that relate *h* ^(*r*)^(***x***, ***u***) to *h*(***x***):

$$\begin{array}{cc} \dot{\eta }\left(x\right) & =F\eta \left(x\right)+G\mu ,\\ h\left(x\right) & =C\eta \left(x\right). \end{array}$$ (15)

With

$$\begin{array}{ll} \eta \left(x\right) & =\left[\begin{array}{c} h\left(x\right)\\ L_{f}h\left(x\right)\\ L_{f}^{2}h\left(x\right)\\ \vdots \\ L_{f}^{r-1}h\left(x\right) \end{array}\right]=\left[\begin{array}{c} h\left(x\right)\\ \dot{h}\left(x\right)\\ \ddot{h}\left(x\right)\\ \vdots \\ h^{\left(r-1\right)}\left(x\right) \end{array}\right],\;\\ \mu & =L_{f}^{r}h\left(x\right)+L_{g}L_{f}^{r-1}h\left(x\right)u=h^{\left(r\right)}\left(x,u\right) \end{array}$$ (16)

and **F** : *r* × *r*, **G** : *r* × 1, **C** : 1 × *r*

$$F=\left[\begin{array}{ccccc} 0 & 1 & 0 & \ldots & 0\\ 0 & 0 & 1 & \ldots & 0\\ \vdots & \vdots & \vdots & \ddots & \vdots \\ 0 & 0 & 0 & \ldots & 1\\ 0 & 0 & 0 & \ldots & 0 \end{array}\right],\;G=\left[\begin{array}{c} 0\\ 0\\ \vdots \\ 0\\ 1 \end{array}\right],\;C=\left[\begin{array}{cccc} 1 & 0 & \ldots & 0. \end{array}\right]$$ (17)

Choosing the state feedback *μ* = −***K*** *η*(***x***) leads to *h*(***x***) being an explicit function of time *t* and the initial state ***x***_0_:

$$h\left(x\right)=Ce^{\left(F-GK\right)t}\eta \left(x_{0}\right),$$ (18)

where *h*(***x***) is then said to be an ECBF—rendering C forward invariant—if there exists a row vector ***K***_*γ*_ such that

$$\underset{u\in U}{\sup}\left[L_{f}^{r}h\left(x\right)+L_{g}L_{f}^{r-1}h\left(x\right)u\right]\geq -K_{\gamma }\eta \left(x\right),\;\forall x\in Int\left(C\right)$$ (19)

results in

$$h\left(x\left(t\right)\right)\geq Ce^{F-GK_{\gamma }}t\eta \left(x_{0}\right)\geq 0,$$ (20)

where *h* (***x***_0_) ≥ 0. A ***K***_*γ*_ can be found that satisfies this using the pole placement method, with all poles being real and negative. Additionally, the eigenvalues *λ*_*i*_ of system **F** − **G*K***_*γ*_ must adhere to a condition based on the initial conditions ***x***_0_:

$$\lambda_{i}\left(F-GK_{\gamma }\right)\geq -\frac{{\dot{\nu }}_{i-1}\left(x_{0}\right)}{\nu_{i-1}\left(x_{0}\right)},$$ (21)

where

$$\begin{array}{ll} \nu_{0}\left(x\right) & =h\left(x\right),\\ \nu_{1}\left(x\right) & ={\dot{\nu }}_{0}\left(x\right)+s_{1}\nu_{0}\left(x\right),\\ & \vdots \\ \nu_{r}\left(x\right) & ={\dot{\nu }}_{r-1}\left(x\right)+s_{r}\nu_{r-1}\left(x\right), \end{array}$$ (22)

and *s*_0_, *…* , *s*_*r*_ are defined as the roots of the characteristic polynomial of **F** − **G*K***_*γ*_.

## 4 Approach

This section presents our proposed method for safe active search under uncertainty applied to ultrasound image acquisition.

### 4.1 Ultrasound image quality optimization

We will now present our proposed solution to Problem 1. In regular Bayesian optimization, sampling from anywhere in the search space is assumed to be associated with the same cost. As the robot must move to acquire images, this assumption is not ideal for robotic ultrasound scanning, as switching between sampling points far apart from each other will incur a large time cost while the robot moves between them. To account for this, we consider a non-standard acquisition function that incorporates the ability to sample ultrasound images throughout the path traveled by the robot. Similar methods have been applied to robotic environmental monitoring using unmanned aerial vehicles (Marchant and Ramos, 2014). We parameterize the path as straight lines to ensure the optimization time of the acquisition function is kept low. The path P is defined as

$$P\left(u,p_{n+1}|p_{n}\right)=\left(1-u\right)p_{n+1}+up_{n},$$ (23)

with *u* ∈ [0, 1]. The modified acquisition function is denoted *α*_*IC*_(⋅) and is said to be informative continuous as information throughout the path is considered. It is defined by integrating the base acquisition function over the path and scaling with the inverse of the path length:

$$\begin{array}{ll} \alpha_{IC}\left.\left(P\left(u,p_{n+1}|p_{n}\right)\right),\alpha \right) & =\frac{1}{\|p_{n+1}-p_{n}{\|}_{2}}\\ & \int_{0}^{1}\alpha \left(P\left(u,p_{n+1}|p_{n}\right)\right)du. \end{array}$$ (24)

The scale is included to account for the time spent moving between ***p***_*n*_ and ***p***_*n*+1_. Assuming that the robot moves at a constant velocity, maximizing the modified acquisition function leads to a path that maximizes the integral of the base acquisition function per unit of time spent. The formulation leads to the entire path being considered—and thus less time spent in areas with low uncertainty and/or low expected scores. We employ the EI acquisition function as the base acquisition function and denote the informative continuous version as ICEI. A score *y* is associated with each position in the search space ***p*** using a classifier trained on labeled ultrasound images. The range of the output is [0; 1], with 0 indicating poor quality and 1 good. Defining an appropriate stopping criterion is important to ensure a good area has been found without spending excessive time scanning a patient. A statistical stopping criterion is proposed based on the maximum lower bound over the samples, ***y***. The lower bound for a sample is

$$y_{1-\delta }=y-Z_{\delta }\sigma_{y},$$ (25)

where *Z* is the standard normal distribution, *Z*_*δ*_ is the critical value for a confidence level of 1 − *δ*, and *σ*_*y*_ is the prediction of standard deviation for the corresponding sample *y* based on the surrogate model. As the range of possible scores is known beforehand, defining an appropriate threshold is possible.

### 4.2 Safe direct force control

**FIGURE 2:**
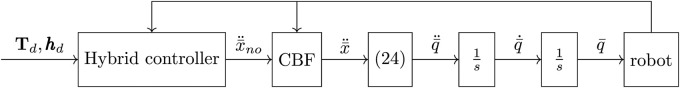
Block diagram of hybrid force/position controller with CBF to ensure bounded contact force. **T**_*d*_ specifies the desired pose, and ***h***_*d*_ specifies the desired wrench.

Our proposed solution to Problem 2 combines a hybrid force/position controller—also referred to as the nominal controller—with a CBF defined such that bounded contact force is ensured. The hybrid force/position controller allows direct control over the force to ensure consistent ultrasound image quality while making positioning possible during contact. The robot considered in this work is controlled at the joint position level. The CBF is defined in Cartesian space and at the acceleration level to allow for constraints on the force. End-effector accelerations are related to joint accelerations using the following kinematic relationship:

$$\ddot{q}=J^{†}\left(q\right)\left(\ddot{x}-\dot{J}\left(q\right)\dot{q}\right),$$ (26)

where **J**(***q***) is the manipulator Jacobian, ^†^ denotes the Moore–Penrose pseudo inverse, ***q*** is the joint configuration, and $\ddot{x}$ is the Cartesian space acceleration. The resulting joint accelerations are then double integrated for the robot’s position controller. Figure 2 illustrates the proposed control architecture.

#### 4.2.1 Hybrid force/position controller

The error between the desired and measured end-effector wrench is denoted Δ ***h***. Similarly, the positional error used is denoted Δ ***x***; note that the orientational error is obtained using quaternions. The Cartesian space is divided such that force control is applied in some subset of the space, and position control is applied in the remainder of the space (Siciliano et al., 2008). New variables are defined to achieve this:

$$\underset{m\times 1}{\Delta h^{*}}=\underset{m\times 6}{S_{f}^{†}}\;\underset{6\times 1}{\Delta h},$$ (27)

$$\underset{n\times 1}{\Delta x^{*}}=\underset{n\times 6}{S_{p}^{†}}\;\underset{6\times 1}{\Delta x},$$ (28)

where $S_{p}^{†}$ and $S_{f}^{†}$ select the position-controlled and force-controlled subsets, respectively. Force control is applied along the length of the probe, defined as the *z*-axis, while positional control is used for the remaining degrees of freedom (DOFs). The force controller acting on a subset of the Cartesian space is then

$${\bar{a}}_{f}=K_{p}^{f}S_{f}^{†}\Delta h-K_{d}^{f}S_{f}^{†}{\dot{x}}_{e},$$ (29)

where ${\dot{x}}_{e}$ is the end-effector velocity, ${\bar{a}}_{f}$ is the resulting acceleration reference, and $K_{p}^{f},K_{d}^{f}$ are diagonal *m* × *m* positive definite gain matrices. Note that ${\dot{x}}_{e}$ is used over the more natural choice of using the end-effector wrench derivative ${\dot{h}}_{e}$ . This is a method of avoiding the problem of taking the time derivative of the often highly noisy force measurements (Siciliano et al., 2008). The position controller is defined similarly as

$${\bar{a}}_{p}=K_{p}^{p}S_{p}^{†}\Delta x-K_{d}^{p}S_{p}^{†}{\dot{x}}_{e},$$ (30)

with ${\bar{a}}_{p}$ as the acceleration reference, and $K_{p}^{p},K_{d}^{p}$ are *n* × *n* positive definite gain matrices. The full acceleration of the nominal controller is then

$${\ddot{\bar{x}}}_{no}=S_{p}{\bar{a}}_{f}+S_{p}{\bar{a}}_{p}.$$ (31)

#### 4.2.2 CBF for bounded contact force

The acceleration reference from the hybrid force/position control is modified by a control barrier function to ensure bounded contact force. This requires the definition of a control affine model relating commanded end-effector accelerations to contact force. This is defined as being one-dimensional, only considering the force from movement along the *z*-axis, thus neglecting friction in the axes of motion. This is a valid assumption, as the US gel placed on the patient’s hand while scanning will render friction between the probe and hand negligible. The contact force is modeled using the Kelvin–Voigt model as

$$f_{c}=k\left(z-z_{r}\right)+b\dot{z},$$ (32)

where *f*_*c*_ is the contact force, *k* and *b* are positive scalars, and *z*_*r*_ is the position of the contact surface.

A second-order system is used to model the inner position control loop and account for the actuator dynamics. The relationship between position reference $\bar{z}$ and actual position *z* is modeled by a second-order system:

$$\ddot{z}=\omega_{n}^{2}\left(\bar{z}-z\right)+2\zeta \omega_{n}\dot{z},$$ (33)

where *ζ* is the damping factor and *ω*_*n*_ is the undamped natural frequency. The control input to the CBF from the nominal force controller is defined at the acceleration level $u=\ddot{\bar{z}}$ . To relate this to $\bar{z}$ , it is double integrated:

$$\bar{Z}\left(s\right)=\frac{1}{s^{2}}\ddot{\bar{Z}}\left(s\right).$$ (34)

The full control affine model is then

$$\begin{array}{c} x=\left[\begin{array}{c} f_{c}\\ z\\ \dot{z}\\ \bar{z}\\ \dot{\bar{z}} \end{array}\right]=\left[\begin{array}{c} x_{1}\\ x_{2}\\ x_{3}\\ x_{4}\\ x_{5} \end{array}\right],\;u=\ddot{\bar{z}}\\ \left[\begin{array}{c} {\dot{x}}_{1}\\ {\dot{x}}_{2}\\ {\dot{x}}_{3}\\ {\dot{x}}_{4}\\ {\dot{x}}_{5} \end{array}\right]=\left[\begin{array}{c} kx_{3}+b\left(\omega_{n}^{2}\left(x_{4}-x_{2}\right)+2\zeta \omega_{n}x_{3}\right)\\ x_{3}\\ \omega_{n}^{2}\left(x_{4}-x_{2}\right)+2\zeta \omega_{n}x_{3}\\ x_{5}\\ 0 \end{array}\right]+\left[\begin{array}{c} 0\\ 0\\ 0\\ 0\\ 1 \end{array}\right]u. \end{array}$$ (35)

Note that this utilizes the derivative of the force, such that the method does not directly depend on *z*_*r*_. However, it will lead to an overly conservative barrier function outside of contact, as the non-contact situation is not modeled. The barrier function is defined as

$$h\left(x\right)=f_{\max}-f_{c},$$ (36)

where *f*_max_ is the maximum allowable force. This leads to a relative degree of three for the system, and an ECBF is therefore employed. The ECBF is defined as a constraint to a quadratic program (QP). The objective function is defined as the squared error between the nominal control input *u*_no_ and the resulting input *u*

$$|u-u_{\text{no}}{|}^{2}=u^{2}-2u_{\text{no}}u+u_{\text{no}}^{2}.$$ (37)

This ensures the nominal control is followed whenever possible, and the ECBF only limits the input whenever the system is near the boundary of the safe set. The resulting QP is then

$$\begin{array}{cc} u^{*}\left(x,u_{\text{no}}\right)=\underset{u\in R}{\arg}\min\; & u^{2}-2u_{\text{no}}u\\ s.t.\; & L_{f}^{r}h\left(x\right)+L_{g}L_{f}^{r-1}h\left(x\right)u+K_{\gamma }\eta \left(x\right)\geq 0, \end{array}$$ (38)

where *u* ^*^ is the modified control input and *u*_no_ is the nominal control input from the hybrid force/position controller.

## 5 Results

In this section, results for the image quality optimization method and safe force controller are presented. The standard EI acquisition function is compared with the proposed ICEI variant. The addition of a CBF for keeping bounded contact force is evaluated under uncertainty in the estimate of surface location *z*_*r*_.

### 5.1 Comparing image quality optimization methods

**FIGURE 3:**
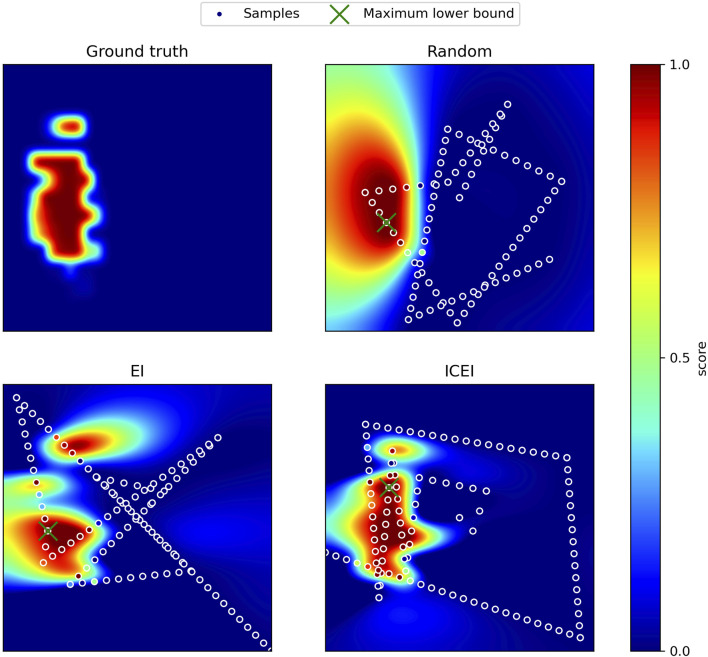
Ground truth and results for random sampling, EI, and ICEI are shown. The predictions of the Gaussian process regression are displayed with the samples and the location of the maximum lower bound.

The ultrasound image quality optimization approach assumes a starting position is given, and predetermined bounds of the search space are defined in relation to this. The search was performed over two DOFs such that the input to the Bayesian optimizer was $p={\left[x,R_{x}\right]}^{T}$ , where *R*_*x*_ is rotation about the *x*-axis. The inherent noise of the measurements was set to *σ*_*ϵ*_ = 0.1. Bayesian optimization was implemented using BoTorch (Balandat et al., 2020). The informative continuous extension was implemented in PyTorch, estimating the integral using a discrete sum. Our proposed method for optimizing ultrasound image quality has been evaluated in a simulated setting. The simulation is based on data gathered from scanning 32 joints across four test subjects. The search space was sampled in a grid using nearest-neighbor interpolation to construct an approximated ground truth. The stopping criterion was set to a maximum lower bound of 0.9 with a confidence level of 95%. Three methods were tested: random sampling, EI, and ICEI. The search space was normalized such that the range was equal for the two DOFs. A distance budget of 10 times the length of the search space was given. In order to simulate the ability to sample continuously, a sample was taken for every 4% of the search space traveled. Based on this, random sampling, EI, and ICEI resulted in 60.7%, 67.8%, and 75.0% success rates, respectively, with mean distance traveled being 8.65, 8.13 and 7.64 times the length of the normalized search space respectively. Figure 3 shows the search patterns resulting from the different methods. Random sampling is, as expected, inefficient. The tendency of ICEI to cause less overlap of paths compared to EI is clear.

EI and ICEI were also compared in a live test. In these tests the search was extended such that $p={\left[x,R_{x},R_{y},R_{z}\right]}^{T}$ with a range of [−7.5*mm*, 7.5 *mm*] for *x* and [−4°, 4°] for the rotational DOFs. The test compared the results of searching using EI and ICEI. For each test, the methods were started at the same position. The methods were run until the stopping criterion was reached. When this occurred, the probe was positioned at the maximum lower bound, and a measurement was taken to ensure the success criterion actually had been reached. This cannot be guaranteed due to uncertainties in positioning. If not, the search was continued. If a solution had not been found within 60 s, it was considered a failed scan. Fourteen joints were scanned. EI lead to a success rate of 79% and ICEI to a rate of 92%. The mean recorded time for optimizing the acquisition function during this test was 0.10 ± 0.06 s for EI and 0.14 ± 0.10 s for ICEI. The training time for the Gaussian process using maximum log-likelihood was 0.16 ± 0.08 s. Even with the ICEI acquisition method, querying a new sample point only took around 0.3 s

### 5.2 Evaluation of safe force controller

A KUKA AG IIWA 7 R800 was used to evaluate the safe controller with the fast research interface (FRI) for communication with the robot through the IIWA ROS package (Chatzilygeroudis et al., 2019). The communication rate with the inner joint position controller was set to 200 *Hz*. The *KYOTO KAGAKU Rheumatism Hand Phantom* was used with parameters estimated to $\hat{k}=1285$ and $\hat{b}=15$ . For the inner joint position controller, the following parameters were found: ${\hat{\omega }}_{n}=31.25$ and $\hat{\zeta }=1$ ; it is not possible to change these parameters, as it is an inbuilt controller. The parameters of the joint controller were estimated by minimizing the sum of squared errors between the model and measurements taken during an initial experiment where the robot was moved into contact with the hand phantom.

The parameter ***K***_*γ*_ used in the safety filter must be defined such that the poles of System (20) are negative and real and adhere to the constraints on initial conditions (21). Pole placement within these constraints affects how conservative the barrier function is and will always ensure safety. The following poles ***p*** were utilized in the experiments: $p=\left[\begin{array}{ccc} -3 & -6 & -9 \end{array}\right]$ . A maximum force of *f*_max_ = 5*N* was specified.

**FIGURE 4:**
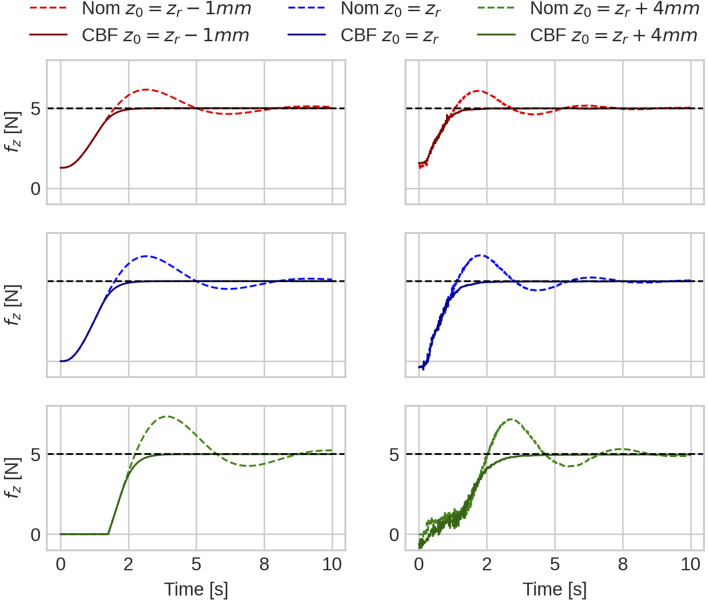
Experiments in simulation (left column) and on the robot (right column) of the robot establishing contact with the environment under initial condition *z*_0_ relative to the surface position *z*_*r*_. The nominal control alone (denoted NOM) does not bound the contact force during contact establishment and leads to varying violations depending on the initial condition. The addition of the proposed CBF ensures contact force is bounded below 5*N* for varying initial conditions ranging from starting 1 mm (*z*_0_ | = *z*_*r*_ − | 1 mm) inside the contact to starting 4 mm above (*z*_0_ | = *z*_*r*_ + | 4 mm) the contact.

The nominal force control parameters where set to $K_{p}^{f}=0.001$ and $K_{d}^{f}=1$ . The controller was implemented in Python using CVXOPT (Andersen et al., 2020) to solve the ECBF QP problem. The end-effector wrench was estimated from the robot’s joint torque sensors using the following kinematic relationship:

$$h_{e}={\left(J{\left(q\right)}^{T}\right)}^{†}\tau_{\text{external}},$$ (39)

where ***τ***_external_ are the measured external joint torques available through the FRI. Figure 4 illustrates the invariance of the proposed method to the contact surface point in the simulation and when running on the robot and shows that the ECBF is able to bound the contact forces below the specified threshold.

## 6 Discussion

Our methodology is applicable to other robotic applications involving safety-constrained active exploration where contact between the robot and an unknown environment is concerned, such as tactile exploration and manipulation. Our proposed formulation for search using Bayesian optimization with ICEI can help model the kind of sampling scheme applicable to robot tactile exploration, that is, where the full travel path of the robot end-effector must be considered. Our approach to the safe bounding of the contact forces using ECBFs is applicable in the case of robots in contact with the environment where force feedback is available. By directly measuring the contact force and considering its first derivative in the contact model, we increase the system’s robustness to uncertainty in the contact boundary, that is, uncertainty in the switching of the environment dynamics.

However, our method has a number of limitations in real-world applications. Although our CBF formulation is robust to uncertainty in the contact boundary, it is not robust to a time-varying boundary. In an ultrasound scanning application, this might occur if the patient, for example, lifted their hand during the scan. In this case, knowledge of the derivative of the contact location would be needed to guarantee safety. Similarly, the Bayesian optimization approach assumes a static function, that is, that the patient’s hand does not move. Nevertheless, small motions can be accounted for by increasing the uncertainty term *σ*_*ϵ*_.

## 7 Conclusion

Methods for safe active exploration of an unknown environment have been presented in this article. Active search under uncertainty is performed using Bayesian optimization, and safety is achieved through the use of control barrier functions. The approach has been applied to autonomous ultrasound scanning of rheumatoid arthritis, which involves finding areas of high ultrasound image quality by positioning the ultrasound probe on the patient. The method has been demonstrated in both a simulated and real setting. An extension to the standard expected improvement, referred to as informative continuous expected improvement, has been proposed. The method has been shown to increase the success rate from 79% to 92%, given a limited time budget of 60 s. Safety in the context of autonomous ultrasound scanning is defined in terms of keeping contact force bounded. We have shown both in simulation and on a robot that, by using a higher-order control barrier function, we are able to bound contact forces during contact establishment despite incorrectly specifying the contact boundary by −1 or +4 mm.

## Data Availability

The raw data supporting the conclusion of this article will be made available by the authors, without undue reservation.
